# The Role of Mitochondrial miRNAs in the Development of Radon-Induced Lung Cancer

**DOI:** 10.3390/biomedicines10020428

**Published:** 2022-02-11

**Authors:** Assiya Kussainova, Olga Bulgakova, Akmaral Aripova, Zumama Khalid, Rakhmetkazhi Bersimbaev, Alberto Izzotti

**Affiliations:** 1 Department of Health Sciences, University of Genova, Via Pastore 1, 16132 Genoa, Italy; assya.kussainova@gmail.com (A.K.); zumama.khalid@edu.unige.it (Z.K.); 2 Department of General Biology and Genomics, Institute of Cell Biology and Biotechnology, L.N. Gumilyov Eurasian National University, Nur-Sultan, Akmola 010008, Kazakhstan; ya.summer13@yandex.kz (O.B.); aripova001@gmail.com (A.A.); 3 Department of Experimental Medicine, University of Genoa, 16132 Genoa, Italy; 4 IRCCS Ospedale Policlinico San Martino, 16132 Genoa, Italy

**Keywords:** radon, microRNA, mitochondrial microRNA, lung cancer

## Abstract

MicroRNAs are short, non-coding RNA molecules regulating gene expression by inhibiting the translation of messenger RNA (mRNA) or leading to degradation. The miRNAs are encoded in the nuclear genome and exported to the cytosol. However, miRNAs have been found in mitochondria and are probably derived from mitochondrial DNA. These miRNAs are able to directly regulate mitochondrial genes and mitochondrial activity. Mitochondrial dysfunction is the cause of many diseases, including cancer. In this review, we consider the role of mitochondrial miRNAs in the pathogenesis of lung cancer with particular reference to radon exposure.

## 1. Radon-Induced Lung Cancer Epidemiology

Lung cancer is a malignant neoplasm that forms in the epithelial cells of the bronchus. Lung tumors have a high risk of invasion and metastasis.

In the structure of oncological diseases in general, lung cancer had a leading position in the number of deaths among men and women in 2020. In terms of the number of cases, lung cancer is in first place among men (15.4%) and in third place among women (8.8%), after breast and colorectal cancer (https://gco.iarc.fr/) (accessed on 26 December 2021).

Lung cancer includes two basic histological types: small-cell lung cancer (SCLC) and non-small-cell lung carcinoma (NSCLC). SCLC stands at 15% of all lung cancers. It is the most differentiated type and it metastasizes faster, mainly to the lymph nodes [1]. Squamous cell lung cancers (SQCLC), adenocarcinoma, and large-cell anaplastic carcinoma (LCAC) are subtypes of NSCLC [2]. Adenocarcinoma is the least aggressive type of tumor that develops mainly in the peripheral bronchus and is mostly found among nonsmokers [3]. SQCLC develops in the main bronchus. Lung cancer usually has no overt signs and symptoms in its early stages. Therefore, the formation of a tumor in the lung is often diagnosed when it is already in the late stages of the disease, which affects the treatment, prognosis, and the survival of the patients [4]. Lung cancer cases are projected to increase by 70% by 2040 (https://gco.iarc.fr/) (accessed on 26 December 2021).

Lung cancer is a multifactorial disease, and various genetic, epigenetic, and environmental variations play a key role in its manner of development. However, smoking is the first environmental risk factor for the development of lung cancer in smokers. The risk of developing a lung tumor depends on the duration of smoking and the number of cigarettes smoked. Cancer can develop among passive and former smokers. In general, it should be noted that a lung tumor can be formed among people who have never smoked or been exposed to tobacco smoke [5].

However, in the case of lung cancer in non-smokers, exposure to radon is found to be a second environmental risk factor [6]. The International Agency for Research on Cancer (IARC) has also recognized radon as a carcinogen and a leading factor in the development of lung tumors in nonsmoking patients [7]. The increased risk of lung cancer is associated with high levels of radon exposure among underground miners [8]. Previously, it was assumed that exposure to radon is only an occupational risk. However, recent studies have shown a significant association between residential exposure to radon and lung cancer [9]. The WHO estimates that radon is responsible for up to 15% of lung cancer cases worldwide (www.who.int) (accessed on 26 December 2021). Residential radon exposure is a major factor in lung cancer mortality in Spain [10]. A study by Lorenzo-Gonzalez et al. confirmed that residential radon exposure increases the risk of lung cancer at concentrations above 50 Bq/m^3^ [11]. In Italy, the overall proportion of lung cancer deaths attributable to radon is about 10%. It is estimated that the majority of radon-associated lung cancer cases occur among smokers (up to 72%) [12]. There is a higher concentration of radon in new buildings compared to older homes in Canada [13]. There are 350 cases of lung cancer and 255 deaths each year in Ireland due to radon exposure [14].

## 2. Cellular Effects of Radon Exposure

There are three uranium isotopes in nature: uranium-238, uranium-235, and uranium-234. Uranium isotopes are radioactive and unstable, which means that they decay and turn into other elements, which are accompanied by the emission of particles.

The most common isotope found in uranium ore is uranium-238. This isotope has a half-life of about 4.47 billion years. Uranium-238 decays into unstable isotopes of thorium-234 with the formation of alpha radiation. Further decay leads to the formation of isotopes of uranium-234, which continue to decay until the formation of a stable isotope of lead-206 [15]. One of the key elements in the decay of uranium is radon-222, a radioactive unstable isotope, the decay of which occurs quite quickly for about 4 days and is accompanied by alpha radiation. The elements that are formed during the decay of radon isotopes are called “daughter products”. Starting with radon, further decay occurs rather quickly (in up to 30 min) with the emission of beta particles [16].

Most of the natural background radiation in the air comes from radon annually. Radon is a gas that enters the biosphere through cracks in the lithosphere and constitutes the Earth’s natural background radiation. When cells are exposed to densely ionizing radiation, such as radon alpha particles, a cascade of molecular and cellular events can occur, eventually leading to the formation of lung and other malignancies. Starting with the deposition of clusters of ionizations and concluding with the development of cancer, that flow may now be outlined. Cellular damage, DNA breakage, accurate or inaccurate repair, apoptosis, gene mutations, chromosomal alteration, and genetic instability are all caused by ionization. However, this can lead to the production of hazardous byproducts. Radon isotopes reach the lungs by inhalation and cause the formation of toxic byproducts. Endogenous radon exposure harms biomolecules.

According to the International Commission on Radiological Protection (ICRP), the relative biological effectiveness (RBE) of radon is 20, which means that, in living tissue, radon is estimated to create 20 times more damage than equal doses of beta or gamma radiation [17]. Thus, endogenous exposure to radon can cause various cytotoxic effects, such as chromosomal aberrations, the formation of reactive oxygen species, cell cycle disturbances, double-strand DNA breaks, and so on [18]. Meenakshi et al., using chromosomal aberrations as a marker of the risk of exposure to radon, showed that the RBE value for radon can be more than 38 [19].

Radon particles can damage cellular components by two mechanisms: linear energy transfer (LET) and the oxidation of cell components by reactive oxygen species (ROS). As they pass through the cell, the movement speed of alpha particles decreases, resulting in more energy releasing per unit of track length, which leads to the damage of cellular components. The path of the particle through the nucleus of the cell crosses many strands of DNA, and the energy released during this breaks the phosphodiester bond, resulting in the formation of double-stranded DNA breaks (DSBs) [20]. This leads to the most cytotoxic lesions caused by radon, and, in the case of defects in the work of the reparative systems, the formation of such breaks can lead to chromosomal instability [21]. Chromosomal instability is not only one of the causes of carcinogenesis but also contributes to tumor adaptation to cytotoxic anticancer drugs [22].

Endogenous factors of a different nature, including ionizing radiation, can trigger DSBs in mtDNA. However, DSBs, which are formed in mtDNA by radiation or ROS, do not lead to the loss of mitochondrial functional activity or cell death. These cells show the ability to survive with the severe loss of mtDNA, as well as a compensatory mechanism, reflected in the increase in the number of mitochondria [23].

The irradiation of cells stimulates mitochondria to increase the production of ROS [24]; therefore, it is mtDNA that is the closest target for their action. Previously, it was argued that mitochondria lack mtDNA repair mechanisms but recent studies report their presence [25]. RAD23A is a protein involved in NER in the nucleus of cells. It has been shown that RAD23A is involved in mitochondria following the induction of oxidative damage to mtDNA [26]. Protein XRCC4, in combination with DNA ligase IV and DNA-dependent protein kinase, is involved in the repair of double-stranded DNA breaks (NHEJ) and has also been found in mitochondria. However, despite the discovery of repair proteins for mtDNA oxidative damage, the mtDNA repair mechanisms themselves remain unknown [27].

Since water is the main component of cells, because of ionizing radiation absorption by the cell, radiolysis of water occurs, which leads to the formation of ROS [28]. Exceeding the level of cellular levels of ROS can cause damage to mitochondrial membranes [29] and, therefore, causes a violation of the membrane potential of mitochondria, which is the main symptom of mitochondrial dysfunction. Mitochondrial dysfunction leads to the release of pro-apoptotic cytochrome C, the activation of caspases-3 and -9, and further cell apoptosis [30].

## 3. Mitochondrial MicroRNAs

Mitochondria are important cellular organelles that support the life of cells by providing them with energy and play a key role in the process of cell death [31]. Mitochondrial dysfunction can cause serious diseases such as diabetes [32], Alzheimer disease, [33], and cancer [34,35]. MicroRNAs play an important role in the pathogenesis of various diseases. For example, by regulating oncogenes and tumor suppressor genes, microRNAs can control the process of carcinogenesis [36].

The proteome of human mitochondria includes more than 1000 proteins [37], of which only 13 are encoded in the mitochondrial genome and are the main components of the electron transport chain (ETC). The rest of the proteins are encoded in the nucleus and imported into the mitochondria [38].

The presence of pre-miRNAs, as well as mature miRNAs, in mitochondria has been reported, raising the possibility of mitochondrial miRNA synthesis. Some pre-miRNA variants seem to be metabolized in the mitochondria and may be used to produce mature miRNAs, which might be active in mitochondrial transcripts or transferred to the cytosol to interact with genomic mRNA. As a result, mitochondrial-processed miRNAs are anticipated to have a role in post-transcriptional gene regulation linked to mitochondrial functions [39].

They play their role in the normal functioning of mitochondria by regulating the mitochondrial genes themselves or by controlling the expression of nuclear transcripts involved in mitochondrial processes. This cluster of regulatory molecules is called mitochondrial miRNAs (mitomiR) [40]. There are three known miRNAs that are mapped in the mitochondrial genome: miR-1974, miR-1977, and miR-1978 [41].

The miRNAs regulate gene expression through association with the RNA-induced silencing complex (RISC). Dicer forms a RISC complex with RNA-binding proteins, TRBP (HIV-1 transactivating response (TAR) RNA-binding protein), PACT, and Ago-2. Ago-2 is the catalytic center of the RISC complex. Mature miRNAs loaded into the RISC complex bind to the 3′-untranslated region (3’-UTR) of mRNA. Furthermore, the translation inhibition or complete degradation of mRNA occurs [42]. Protein GW182 stabilizes the RISC complex by binding to Ago-2 [43]. The interaction of the GW18 protein with Ago-2 is important for the regulation of translation in the cytoplasm. However, for the translocation of microRNA into mitochondria, it is necessary to separate GW182 from the Ago-2 protein. Zhang et al. found that all three members of the GW182 family were excluded from mitochondria, in contrast to Ago-2 [44].

Usually, the transportation of mitochondrial proteins from the cytoplasm is carried out through special, highly specific transport complexes that are located on the outer (TOM40) and inner (TIM23) mitochondrial membranes. However, some proteins avoid granzymes (they lack the N-terminal mitochondrial target sequence) and use channels SAM50 and TIM22 to cross the OMM and IMM, instead of the canonical TOM40–TIM23 pathway. It should be noted that SAM50 has no channel translocase activity [45].

Cellular stress leads to the thermodynamic instability of GW182 and the release of microRNA/Ago-2 from the RISC complex. The proposed mechanisms for the translocation of the free microRNA/Ago-2 complex into mitochondria include both the TOM20–TIM and SAM50–TIM pathways [46]. However, it remains unknown what mechanism provides the transport of miRNAs into mitochondria.

## 4. The Role of MicroRNA in the Functional Activity of Mitochondria

**Figure 1:**
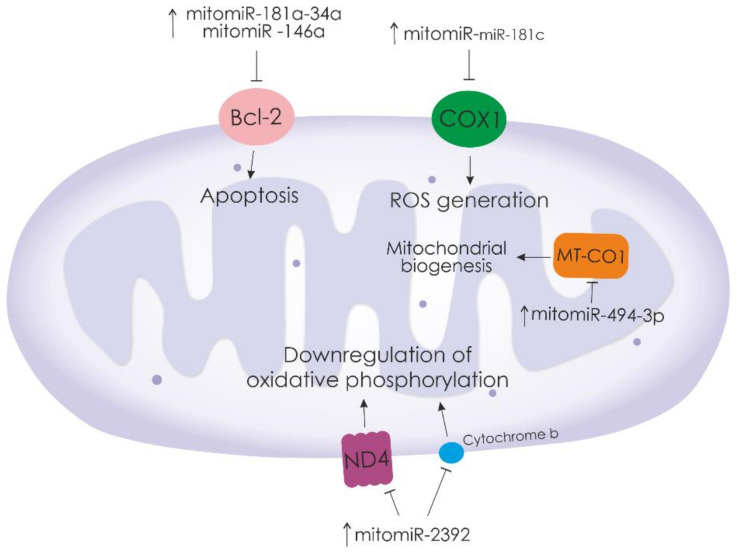
Mitochondrial miRNAs that regulate the expression of major mitochondrial proteins. “
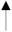
”—activation of downstream signaling pathways. 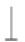
—inhibition of downstream signaling pathways.

Studies have shown that many different miRNAs play a key role in the regulation of mitochondrial functional activity (Table 1, Figure 1). There are identified microRNAs that are involved in the expression of mitochondrial proteins in the cell cytoplasm and mitochondrial matrix [47].

The regulatory role of mitomiR in the mitochondrial metabolism is known. Thus, miR-194 plays an important role in glucose metabolism and is associated with the progression from insulin resistance to type 2 diabetes. Latouche et al. showed that miR-194 is suppressed in the skeletal muscle of insulin-resistant rats, mice with high fat levels, and people with prediabetes and type 2 diabetes [60]. The important role of miR-33 is known in the regulation of lipoprotein metabolism and associated disorders, including metabolic syndrome, obesity, and atherosclerosis [61]. The miR-128-1, miR-148a, miR-130b, and miR-301b control the expression of key proteins involved in cholesterol and lipoprotein transfer, such as the low-density lipoprotein (LDL) receptor (LDLR) and cholesterol transporter ATP-binding cassette A1 (ABCA1) [62]. Changes in mitomiR expression can have a protective effect in the event of cell damage by oxidative stress. Thus, miR-21 mediates a pronounced myocardial protective effect of kaempferol against damage caused by hypoxia and reoxygenation by reducing oxidative stress and activating the Notch1/PTEN/Akt signaling pathway [63]. The overexpression of miR-140 reduces ischemia–reperfusion damage to the myocardium and can also inhibit the expression of Drp1 and Fis1 proteins, which provoke mitochondrial division [64]. The increased expression of miR-200a-3p in proximal renal tubular epithelial cells (TECs) stimulates mitochondrial antioxidant protection and ATP production through the activation of the Keap1–Nrf2 signaling pathway that protects TECs from oxidative effects by normalizing the mitochondrial membrane potential and increasing the mitochondrial DNA copy number [65]. The exposure of HT22 cells to hydrogen peroxide leads to changes in the mitochondrial morphology and dynamic. The administration of exogenous miR-125b decreases cell viability and mitochondrial damage. Against the background of a decrease in miR-125b, an increase in the level of the p53 protein was noted, which led to mitochondrial damage and cell apoptosis. Thus, it was shown that the protective effect of miR-125b is due to the direct targeting of p53 [66].

The role of mitomiR in the regulation of apoptosis is known. For example, the protective properties of miRNA-214 result from a decreased apoptosis of cardiomyocytes in myocardial infarction in the elderly. Four target genes for miRNA-214, PUMA, PTEN, Bax, and caspase-7 were identified, the suppression of the expression of which leads to the inhibition of apoptosis [67]. In addition, an increase in the level of miRNA-214 was observed with a prolonged exposure to 2% sevoflurane, which led to the degeneration of brain neurons and the disruption of mitochondrial morphology. Furthermore, miRNA-214 suppresses the expression of the Mfn2 protein. The decrease in miRNA-214, in turn, led to the activation of the interaction between Mfn2 and Pkm2, which promotes the mitochondrial fusion and decreases brain damage [68].

## 5. The Role of Mitochondria in Oncogenesis and Tumor Development

Cancer is recognized as a multifactorial disease; this definition is consistent with the theory of somatic mutations in tumor initiation. However, there is evidence that cancer is a metabolic disease resulting from disturbances in the metabolic activity of mitochondria. As Warburg suggested, it is the change in mitochondrial respiration that leads to the malignant transformation of cells [69]. However, there is a possibility that the so-called Warburg effect is just a phenotype encoded by the nuclear genome. To determine the possible effect of cytoplasm on oncogenesis, several different experiments have been carried out. Defects in mitochondrial function have long been suspected of playing a role in cancer genesis and progression. All the experiments carried out with the development of cybrid systems clearly showed that tumor development depends on the cytoplasm and cellular components and not the cell nucleus [70,71,72].

Modern research has shown more than once that it is the disturbances in the functional activity of mitochondria that can lead to the malignant transformation of cells. In general, five possible pathways for the participation of mitochondria in carcinogenesis and the further development of the tumor can be identified. First, as we stated earlier, a change in cellular metabolism is one of the causes of malignant cell transformation. Many studies have confirmed this, identifying disorders of mitochondrial metabolism as the cause of cancer development [73,74].

The second cause of carcinogenesis is oxidative cellular stress. A result of metabolism is that mitochondria produce free radicals, which are subsequently capable of attacking the cell. In the process of energy synthesis, the last electron leaving the electron transport chain is oxygen O2, which is further reduced to water. However, about 2% of molecular oxygen is reduced to superoxide, which, in turn, is the precursor to many forms of ROS [75]. Moreover, although ROS are normal products of cellular respiration and are always present in the cell, an increase in their amount, for example, because of the disturbances in mitochondria, leads to damage to cellular organelles and DNA [76]. In addition, the active production of ROS is closely related to the effect of external factors on cells, such as radon and asbestos [32]. It is now evident that ROS induction is associated with the development of cancer at different stages [77], metastasis, progression, and the survival of tumor cells [78,79].

Third, the appearance of a tumor is associated with the suppression of apoptosis. In a way, mitochondria and ROS play the role of a two-faced Janus in carcinogenesis. As a result of overload in the mitochondrial electric transport chain, there is an increased formation of free radicals and ROS, which, in some cases, can lead to carcinogenesis; in others, ROS are effectors for activating the mitochondrial apoptotic pathway. ROS damage the lipid bilayer of the outer mitochondrial membrane, resulting in the release of an apoptogenic protein (CytC) into the cytoplasm. In the cytosol, CytC participates in the assembly of the apoptosome, which activates the initiator caspase-9 and effector caspase-3, leading to cell death [80]. Apoptosis suppresses tumorigenesis by removing damaged cells from the healthy pool. However, cancer cells can inhibit apoptosis, leading to tumor growth. A whole family of apoptosis inhibitor proteins (IAPs) is known, among which IAP is a potent inhibitor of caspases [81]. Many studies show increased IAP expression in various types of cancer, such as cancers of the lung [82], prostate [83], chest [84], bile [85], ovaries [86], and bladder [87], as well as melanoma [88], lymphoma [89], glioblastoma [90], and hepatocellular carcinoma [91]. It is noted that the overexpression of XIAP promotes the survival of cancer cells and the development of cancer [92]; however, mitochondria, in this case, may have a protective function. In response to apoptogenic stimuli, the ARTS protein is released from mitochondria and binds to XIAP, thereby promoting apoptosis [93].

Mitochondria are organelles that are unique in nature. As discussed above, the role of mitochondria in the cell is quite diverse, from providing energy to cell death. Since mitochondria are autonomous organelles with their own ribosomes and DNA, the D-loop region of mitochondrial DNA (mtDNA) contains important regulatory elements, and mutations in this region can lead to certain consequences for the cell. Moreover, the localization of mtDNA in the immediate vicinity of the source of the formation of free radicals and ROS makes it the most vulnerable to damage. Thus, it was studied that somatic mutations of the Mnl I site can be the cause of the pathogenesis of breast cancer [94]. The somatic deletion (50 bp) of the mtDNA D-loop was found in gastric adenocarcinoma [95]. A total of 48 mutant D-loop sites were found from the analysis of brain cancer samples [96]. Three D-loop mutation hotspots at sites 146, 152, and 186 are associated with the risk of oral cancer [97]. Thus, we can conclude that the mutation in mtDNA is the fourth variant of oncogenesis. There is also evidence that not only are mutations in mtDNA involved in the process of cell transformation, but also in changing the number of free circulating mtDNA in the blood plasma of healthy people and cancer patients. Moreover, CS-mtDNA is a molecular marker of oncology [98,99].

Given the participation of mitochondria in apoptosis, it is valid to assume that they are a therapeutic target in the treatment of cancer [100,101]. Nevertheless, mitochondria in this case also exhibit opposite functions and provide cancer cells with drug resistance, which can be attributed to the fifth variant of the participation of mitochondria in the development of cancer. For example, it is known that the overexpression of the mitochondrial protein OPA 1 leads to resistance to cisplatin treatment. At the same time, the suppression of OPA 1 correlates with a high release of cytochrome c into the cytoplasm and promotes apoptosis. Accordingly, the suppression of OPA 1 expression can reduce cisplatin resistance [102]. Another mechanism for the survival of cancer cells after chemotherapy is the transfer of mitochondria; Moschoi R et al. showed this well in their research. Acute myeloid leukemia cells can capture mitochondria from a healthy environment after being treated with cytarabine, which leads to their survival [103].

## 6. The Role of Mitochondrial miRNAs in Oncogenesis and the Development of Various Forms of Cancer

Due to their diverse functions, mitochondria are involved in various mechanisms of cell stress, malignant transformation, and the adaptation of cancer cells, ensuring their survival and tumor development. However, many mitochondrial processes can be controlled and regulated by microRNA molecules. As mentioned above, mitomiR is a separate group of microRNA molecules that can regulate the expression of mitochondrial proteins encoded by nuclear genomes or penetrate mitochondria and control the main regulatory elements of the mitochondrial genome [39].

The miR-1 is an oncosuppressive miRNA; miR-1 expression is known to be suppressed in various types of cancer. A decrease in miR-1 has been noted in prostate cancer. The suppressive function of miR-1, in this case, was achieved by the inhibition of E2F5 and PFTK1, which promote cell proliferation and cell cycle progression [104]. The overexpression of miR-1-3p significantly suppressed proliferation and induced the apoptosis of hepatocellular carcinoma cells by inhibiting SOX9, which reduced tumor volume in vivo [105]. The miR-1 may suppress tumor growth and metastasis in breast and stomach cancers by acting on six target genes (CDK4, TWF1, CNN3, CORO1C, WASF2, and TMSB4X) [106]. Liu C. et al. identified miR-1 as mitomiR because the overexpression of miR-1 caused mitochondrial damage in melanoma and breast cancer stem cells. The miR-1 binds to 3HTO MINOS1, which is a component of the MICOS complex of the inner mitochondrial membrane, maintains the structure of the inner membrane, and participates in the formation of contact sites with the outer membrane, GPD2 (the protein encoded by this gene is localized on the inner mitochondrial membrane and catalyzes the conversion of glycerol-3-phosphate to dihydroxyacetone phosphate), and LRPPRC (in the mitochondria, it binds to mRNA poly (A) and participates in the regulation of transcription). Interestingly, in normal cancer cells, miR-1 activity did not result in mitochondrial destruction [107].

There was a decrease in miR-29b-3p expression in gastric cancer cells compared to normal epithelial cells of the human gastric mucosa. Myc-associated zinc finger protein (MAZ) has been identified as a direct target for miR-29b-3p. The suppression of MAZ led to the inhibition of the proliferation and migration of cancer cells [108]. The miR-29b-3p also inhibits angiogenesis by binding to transcripts of VEGFA and PDGFB genes in endothelial cells of retinal microvessels [109]. Mao A. et al. observed that miR-29b-3p enhances the therapeutic effect of X-ray and radioactive carbon ion irradiation in the treatment of prostate cancer. The miR-29b-3p negatively regulates the expression of WISP1 and, as a result, the anti-apoptotic protein Bcl-XL is suppressed and the mitochondrial apoptosis pathway is triggered. It should be noted that a decrease in miR-29b-3p levels leads to cancer radioresistance [110].

Several studies reported an association between miR-155 and lung cancer [111], esophageal cancer [112], and breast cancer [113]. Changes in the metabolism of cancer cells contribute to the transformation, survival, and invasion of tumor cells. A study by Kim S. et al. showed how oncomiR-155 promotes breast tumor growth by regulating glucose metabolism. The miR-155, through the regulation of the p85α–AKT–FOXO3a pathway, controls cMYC, which is a major regulator of glycolysis. The c-MYC activates glucose transporter proteins and its metabolic enzymes HK2, PKM2, and LDHA. As is known, cancer cells change their metabolism by receiving energy through glycolysis, and miR-155 increases glucose uptake and enhances glycolysis, thereby ensuring the viability of cancer cells [114].

The miR-181a-5p also contributes to the change in glucose metabolism in liver cancer. The overexpression of mitomiR-181a-5p decreases the level of protein components of the electron transport chain mt-CYB and mt-CO2, which results in the decrease in the membrane potential of mitochondria. However, the expression of HK2 (glycolysis regulator) and GLUT1 (glucose transporter) increases. As a result, the increased consumption of glucose and the activation of glycolysis contribute to the proliferation and metastasis of cancer cells [115].

It is known that miR-145 can suppress cell proliferation and migration in melanoma [116] and endometrial cancer [117] as well as stomach cancer [118]. According to a study by Zhao Sh. et al., it can be concluded that miR-145 can be attributed to mitomiR because this mitomiR suppresses the function of mitochondria in ovarian cancer cells. First, miR-145 overexpression correlates inversely with ATP and mtDNA levels in cancer cells due to ARL5B targeting. Second, an increase in the level of miR-145 leads to a decrease in the membrane potential of mitochondria and promotes the release of cytochrome C into the cytoplasm. Thirdly, miR-145 inhibits SDHA (one of the main components of the mitochondrial respiratory chain complex) and HSP60 (a chaperone involved in the correct folding and import of mitochondrial proteins) [119].

The miR-125b is known as a tumor suppressor in breast cancer [120], ovarian cancer [121], hepatocellular carcinoma [122], and esophageal cancer [123]. Xie X. et al. reported that the decreased expression of miR-125b resulted in cell resistance to doxorubicin. A normal miR-125b level increases cell cytotoxicity to doxorubicin and activates cell apoptosis by inhibiting anti-apoptotic protein Mcl-1. The treatment of cells with miR-125b and doxorubicin causes a loss of the mitochondrial membrane potential, which ultimately leads to an increase in the permeability and activation of caspases [124].

Chen W. et al. determined that a decreased expression of mito-miR-5787 leads to chemoresistance to cisplatin by altering glucose metabolism and the negative regulation of MT-CO3. MT-CO3 is a component of cytochrome-c-oxidase of the OXPHOS system, and its suppression weakens OXPHOS. Cytochrome-c-oxidase is a component of the respiratory chain that catalyzes the reduction of oxygen to water, which explains why miR-5787 knockdown increases ROS generation. Moreover, miR-5787 knockdown increases the expression of HK2 and PKM2, which contributes to the increase in glucose uptake and, therefore, to an increased production of lactate. This change in the pH of the cell balance leads to the resistance of the cells to cisplatin [125].

Thus, based on a review of the available information on this subject, it can be concluded that mitomiR can not only be biological markers of oncology but also participate in the survival and development of cancer cells, controlling mitochondrial functional activity.

## 7. The Role of Mitochondria and mtDNA in the Development of Lung Cancer

According to GLOBOCAN data in 2020, out of 36 types of cancer in 185 countries, lung cancer is the most commonly diagnosed form and the leading cause of cancer death among men in 96 countries. Among women, lung cancer is the second most common and the leading cause of cancer death (https://gco.iarc.fr/) (accessed on 26 December 2021).

Although most of the data report that lung cancer is a multifactorial disease, the pathogenesis of which is caused by a genetic predisposition (polymorphisms in the genes for DNA repair and the detoxification of xenobiotics, the spontaneous inactivation of tumor suppressor genes) [126] and exposure to external factors, such as smoking, asbestos [127], and radon [128] in the case of lung cancer.

There are data that show the different roles of mitochondria in the emergence and development of lung tumors [129]. An increase in glucose uptake by cells leads to the synthesis of insulin, which increases the IGF-1 level and suppresses the production of IGF-binding proteins. Typically, tumor cells, including lung cancer cells, have a high level of IGF-1R expression. The IGF-1/IGF-1R complex increases the expression of VEGF, which promotes angiogenesis and tumor development. The knockdown of the SNCG gene leads to the inhibition of IGF-1 and IGF-1R, and, consequently, cell proliferation and tumor growth [130].

It is worth noting that mitochondrial damage leads to tumor progression. Lung cancer metastases showed a lower membrane potential with reduced mitochondrial functionality compared to non-metastatic primary tumors. Electron microscopy of the metastases showed irregular shapes of mitochondria in which transverse bridges were formed between the membranes, which led to a change in the mitochondrial structure [131].

With the growth of lung tumors, mtDNA depletion is also observed, which was associated with a low metastatic potential and an unfavorable prognosis of survival for patients with lung cancer [132]. However, as for freely circulating mtDNA, it is worth noting that in the case of exposure to aggressive factors, such as tobacco smoke, radon, or asbestos, an increase in the level of cf-MtDNA and proinflammatory cytokines in the plasma of patients with NSCLC occurs, which correlates with the poor prognosis of the survival rate [133]. It was noted that cf-mtDNA levels increased in patients with radon-induced lung cancer and in healthy donors living in areas with high radon levels [134]. This can be explained by the fact that the main provocateurs of lung tissue carcinogenesis (smoking and radon) increase the production of ROS, which, in turn, damages various structural parts of cells including the lipid bilayer of mitochondrial membranes. Such damage to mitochondria causes disturbances in their functional activity, affecting the work of the electron transport chain and the oxidation of mtDNA. In response to oxidative stress, mitochondria seek to increase the number of copies of their DNA [135]. Long-term exposure to negative factors contributes to the constant production of ROS, which leads to greater consequences: the apoptosis [136] or necrosis [137] of cells.

In the case of apoptosis, the BAX (Bcl-2-associated X protein) and BAK (BCL2 Antagonist/Killer 1) proteins invading the mitochondrial membrane increase its penetrating ability. As a result, intracellular mtDNA released into the cytosol can be associated with cGAS–STING, TLR9 (Toll Like Receptor 9), and the inflammasomes NLRP3 (NOD-, LRR- and pyrin domain-containing protein 3), NLRC4 (NLR Family Pyrin Domain Containing 4), and (absent in melanoma 2), which promotes an enhanced immune response [138]. Similar to bacterial DNA, mitochondrial DNA is hypomethylated at CpG islands, which explains its recognition by TLR9 receptors [139]. After binding to mtDNA, TLR9 recruits its intracellular adapter MyD88, which activates a number of signaling proteins such as MAPK and NF-κB [140]. NF-κB is a central participant in many inflammatory processes; after activation, it is translocated into the nucleus and, acting as a transcription factor, triggers the synthesis of pro-inflammatory cytokines and chemokines [141]. However, genes activated by NF-κB can be involved in various processes of cell carcinogenesis. For example, anti-apoprotein, Fas, BCL-2, and c-FLIP promote cell survival and avoid apoptosis; BCL-2L1, PAL2 b, and cyclins regulate cell proliferation; cytokines IL-1, IL-2, IL-6, IL-8, IL -12, and TNF-a support inflammation; chemokines MCP-1, IL-18, CXCL1m and CXCL10 cause angiogenesis; and ICAM-1, VCAM-1, and ECAM-1 promote invasion and metastasis [141]. Dimitrakopoulos et al. reported that the dysregulation of NF-κB in NSCLC plays a negative role in predicting patient survival and increases the chance of disease recurrence [142]. It is known that mtDNA can move to neighboring cells to spread the inflammation focus. Horizontal transfer of mtDNA is carried out using several possible mechanisms: directly through gap junctions between cells, nanotubules formed between neighboring cells in the tumor microarray, or packing into extracellular vesicles [143]. Maintaining inflammation is very important for the onset and development of lung cancer. COPD is known to be associated with chronic inflammation and is a precancerous disease. The mtDNA profile was significantly increased in both COPD and lung cancer [144]. However, inflammation can express a protective effect against tumors. After activation, neutrophils create antitumor immunity, which is aimed in suppressing cancer [145]. A low index of inflammation is associated with poor overall survival of cancer patients with NSCLC and squamous cell carcinoma of the head and neck as well as with diffuse large-cell lymphoma [146]. Prolonged systemic inflammation results in mtDNA being released into the bloodstream, stimulates the neutrophil activity through TLR9 binding and p38 MAPK activation, and causes inflammation [147]. Lai et al. determined that cc-mtDNA can be identified as a diagnostic biomarker for NSCLC. In addition, different levels of cytokine expression induced by TLR9 activation can be an important criterion for determining the stages of cancer and making a prognosis for lung cancer patients [148].

Since mitochondria are generators of ROS, it is mtDNA that is the closest target for their action. As a result, various mutations accumulate in mtDNA. The mtDNA mutations are common in lung cancer. Raghav et al. found that the mean germline mtDNA mutation rate in stage 1 lung adenocarcinoma was 2.29 mutations per kbp. Most of the mutations occurred in the D-loop and the CYTB gene (which are part of the mitochondrial respiratory chain) [149].

## 8. Mitochondrial miRNAs Are Involved in the Development of Lung Cancer through the Regulation of Mitochondrial Genes

It is impossible to answer the question of how exactly a tumor arises and develops. There are many mechanisms underlying carcinogenesis. This is a rather complex process that includes several successive stages necessary for the full growth of the tumor. Intracellular changes or cell damage initiates the process of the malignant transformation of cells. Furthermore, there is a change in the phenotype of cancer cells, cells stop responding to cellular signals and lose control over proliferation, and the tumor begins to actively grow and form its own microenvironment. After the formation of a non-invasive tumor, which is also called “cancer in place”, angiogenesis begins, new blood vessels are formed, which directly feed the growing tumor, and the growing tumor progresses [150].

Each stage from the initiation of tumorigenesis to metastasis is accompanied by various biochemical changes and is initiated by cellular signals. For example, the oxygen starvation of a tumor activates HIF-1α, which, in turn, induces the expression of VEGF and its receptor, which eventually stimulates the formation of new blood vessels [151].

MicroRNA molecules can regulate any pathway of cell signaling and, accordingly, they can control the stages of carcinogenesis [152]. The role of microRNA in the development of radon-induced lung cancer as well as asbestos-induced lung cancer is already known [153]. Therefore, it is of interest to consider the role of mitomiR in the pathogenesis and development of lung cancer.

Transcription factors also play a vital role in cancer proliferation; the overexpression of the transcription factor NFAT5 promotes the proliferation and migration of lung adenocarcinoma cells [154]. Phosphoglycerate kinase 1 (PGK-1)*,* one of two ATP-generating enzymes during glycolysis, is the target gene for NFAT5 [155]. The miR-194 suppresses the migration and proliferation of NICRL cells, inhibiting the expression of NFAT5, whereas the level of miR-194 decreases in NSCLC cells with a high content of glucose [156].

Oxidative stress induces the expression of miR-200c, which leads to cell death. The transcriptional repressor ZEB1 is a direct target for miR-200c [157]. Decreased ZEB1 expression in NSCLC correlates with overall patient survival. The lower the expression of ZEB1, the better the survival rate with NSCLC [158]. MaD’Almeida et al. studied the effect of miR-200c encapsulated in cholesterol carriers in the cells. Given the nature of the carrier, the Nano miR-200c was designed to penetrate the mitochondria and the cell nucleus. As a result, an increase in the expression of mitochondrial genes was observed, which can later be used to restore the membrane potential of mitochondria and normalize the work of the electron transport chain (ETC) [159].

The miR-214 is an oncosuppressive agent in the advanced types of cancer; however, studies show it to be an inducer of cell proliferation and metastasis in lung cancer. The miR-214 regulates hypoxia-inducible factor 1 (HIF-1α), matrix metalloproteinase-2 (MMP2), and vascular endothelial growth factor (VEGF) by inhibiting the inhibitor of growth protein 4 (ING4) [160]. The suppression of miR-214 expression reduces the rate of glycolysis and lactate production by decreasing the level of hexokinase-2 (HK2), which plays a key role in maintaining the integrity of the outer mitochondrial membrane and pyruvate kinase (PKM2) in NSCLC cells. The miR-214 is capable of increasing cell proliferation by targeting PTEN and, consequently, regulates the PI3K/AKT/ mTOR signaling pathway [161].

## 9. Effect of Radon on Both MicroRNA Profile and Lung Cancer Risk

MicroRNAs regulate a variety of cellular processes, including those induced by the effects of radiation exposure. Several studies recognize microRNAs as biomarkers for assessing the degree of radiation contamination with radon. They are important regulators of various genes associated with the risk of lung cancer.

Sun et al. identified miR-19a, miR-30e, miR-335, and miR-451a as potential biomarkers of radiation damage by radon. These microRNAs were reduced in the peripheral blood of miners who had been exposed to radon for a long time, whereas the level of cyclin A2, cyclin D1, and cyclin E1 was significantly increased [162].

Several studies have been performed using the BEAS-2B healthy cell line model, and long-term exposure to radon led to an increase in miR-34a expression in it. As a result, the levels of the anti-apoptotic proteins Bcl-2 and PARP-1 were decreased, and the expression of the pro-apoptotic protein Bax was increased. As a result, an increase in the apoptosis of BEAS-2B cells was observed [163]. Cui et al. conducted a study of the change in the levels of microRNA in cells after irradiating with a high dose of radon (20,000 Bq/ m3) five times. Screening for differential miRNA expression was performed after the first passage (Rn5-1 cells) and after the 20th passage (Rn5-20 cells). As the outcome, 163 increased and 155 decreased Rn5-1163 cells were found. Rn5-20 cells had 30 upregulated and 28 downregulated miRNAs [164]. Dang et al. studied the effect of radon alpha radiation on the microRNA profile, using cell models with different radiation doses of 0.1, 0.5, and 2 Gy; a total of 24 and 41 miRNAs showed a change in the microRNA profile in the 10th and 40th generations of cells, respectively [165] (Table 2, Table 3).

Another study showed a decrease in the microRNA profile (hsa-miR-16-2-3p, hsa-miR-182-3phsa-miR-221-5p, hsa-miR-30c-2-3p, hsa-miR-3660, hsa-miR-4306, hsa-miR-4440, hsa-miR-4443, hsa-miR-452-5p, hsa-miR-454-3p, hsa-miR-455-3p, hsa-miR-4793-3p, hsa-miR-598-3p, hsa-miR-6500-5p, hsa-miR-6826-5p, hsa-miR-6872-3p 0, hsa-miR-7159-5p, hsa-miR-98-5p) of the patients with diagnosed lung cancer who live in areas with high levels of radon in the air [166].

**Figure 2:**
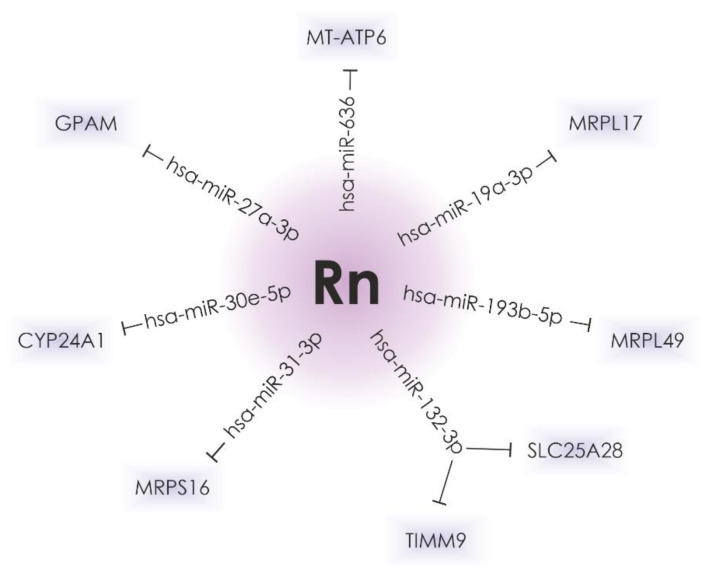
The hsa-miR-636, hsa-miR-27a-3p, hsa-miR-30e-5p, hsa-miR-31-3p, hsa-miR-132-3p, hsa-miR-193b-5p, and hsa-miR-19a-3p, the expression of which is significantly altered in lung cancer and after radon irradiation, can possibly regulate the expression of genes that encode mitochondrial proteins.

Possible microRNA target genes whose expression is affected by radon exposure were predicted using TargetScan software (Release 7.2). A total of seven of them are presumed to play a role in mitochondrial protein expression (Figure 2). These data can be confirmed by further experiments.

Alpha radiation is able to activate autophagy and promote ROS accumulation by upregulating miR-22. Regulated in development and DNA damage response 1 (REDD1) is known as a gene that is activated in response to DNA damage [167]. REDD1 has been identified as a direct target gene for miR-22. REDD1 accumulates in mitochondria and serves as a regulator of mitochondrial metabolism. During irradiation, miR-22 blocks the expression of REDD1, thereby promoting the accumulation of DNA damage and active ROS generation and enhancing apoptosis [168].

In mammals, one important epigenetic mechanism for regulating chromatin structure and gene expression is DNA methylation [169]. Many CpG sites regulate miRNA expression in cells. Changes in DNA methylation can lead to the differentiated expression of miRNA in cancer cells [170]. Hypermethylation in p16INK4 and O6-MGMT is proportional to the effect of radon among uranium miners. However, ionizing radiation above 1 Gy is often characterized by a loss of global DNA methylation [171]. If ionizing radiation and radon, among other things, can influence methylation patterns, then it is possible this causes the alteration of microRNA expression after exposure to radiation. It is possible that hypomethylation leads to overexpression, and hypermethylation, on the other hand, reduces the microRNA profile.

## 10. Conclusions

Radon-induced lung cancer is a significant problem worldwide. Radon is the main component of the radioactive background of our planet. The population is exposed to ionizing radiation at home and in the workplace. Despite the fact that the WHO has determined the permissible radon standards to be 100 Bq/m3, the risk of lung cancer increases from 50 Bq/m3. Prolonged exposure to radon causes damage to cellular components, mitochondria, and oxidative stress, which leads to damage to lung tissue [172]. The extensive role of microRNA molecules in the regulation of carcinogenesis processes induced by radon has long been known. MitomiR is a class of microRNA molecules that can regulate the expression of mitochondrial proteins and control the functional activity of mitochondria. Therefore, it was of interest to investigate the participation of mitomiR in the development of lung cancer. In our review, it was shown that various mitomiRs are involved in the pathogenesis and progression of lung cancer acting as regulators of mitochondrial processes. As a result, it is possible to state the study of mitomiRs as new biomarkers of radon-induced lung cancer is a relevant and promising area.

## Figures and Tables

**Table 1:** The role of various miRNAs in the regulation of mitochondrial genes.

mitomiR	Up/Down	Effects	Target Gene	Localization	Link
**miR-181a-34a and -146a**	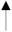	Induces mitochondrial function, activates pro-apoptotic and pro-inflammatory caspases, and promotes apoptosis.	*Bcl-2*	Can be localized to and enriched in the mitochondria of sHUVECs	[48]
**miR-181c**	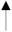	Changes in mitochondrial metabolism and high ROS generation.	3′-end of *mt-COX1*	Mitochondria Cytoplasm	[49]
**miR-2392**	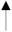	Decrease in oxidative phosphorylation and increased glycolysis.	mtDNA at region (4379–4401), *ND4, CYTB, COX1*	Mitochondria	[50]
**miR-378a-3p**	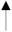	Cell apoptosis	*PDK1*	Cytoplasm	[51]
**miR-107**	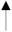	Inhibition of β-oxidation in hepatocytes. Local accumulation of lipids. Impaired glucose tolerance.	*HADHA*	Cytoplasm	[52]
**miR-494-3p**	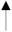	Regulation of mitochondrial biogenesis in adipocytes.	*PGC1-α, TFAM, PDH, Ucp1,* and *CIDEA* *MTCO1*	Cytoplasm Mitochondria	[53]
**miR-410-3p**	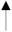	Reduces mitochondrial membrane damage and mitochondrial swelling.	*TLR2*	Cytoplasm	[54]
**miR-210**	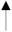	Changes in energy metabolism. Induces oxidative stress.	*ISCU*	Cytoplasm	[55]
**miR-141-3p**	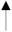	Neurotoxicity, apoptosis, and potential abnormalities in mitochondrial membranes.	*SIRT1*	Cytoplasm	[56]
**miR-574-5p or miR-574-3p**	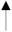	Regulation of the protein expression of mitochondrial electron transport chain (ETC) genes.	*FAM210A, MEG*	Cytoplasm	[57]
**miR-204-5p**	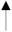	Increases oxidative capacity and mitochondrial degradation.	*PGC-1α*	Cytoplasm	[58]
**miR-762**	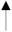	Could contribute to inhibiting ATP production and inducing ROS generation and apoptotic cell death.	*CDS* of mitochondrial *ND2*	Mitochondria	[59]

**Table 2:** The miRNAs that were significantly increased after exposure to different concentrations of radon in cells.

**Cellular Model of Irradiation**	**«2B-2.0 Gy (4) -40»** [161]	**«Rn5-1 Cells (20,000 Bq/m3)»** [160]	**«Rn5-20 Cells (20,000 Bq/m3)»** [160]
MicroRNAs whose profile has been altered by radioactive irradiation	hsa-miR-550b-2-5p, hsa-miR-3907, hsa-miR-224-3p *, hsa-miR-6512-5p, hsa-miR-195-5p */**, hsa-miR-652-3p */**, hsa-miR-6516-3p, hsa-miR-132-3p **, hsa-miR-5001-5p, hsa-miR-8072, hsa-miR-4257, hsa-miR-5787, hsa-miR-4646-5p, hsa-miR-5001-5p	hsa-miR-16-5p, hsa-miR-15b-5p */**, hsa-miR-15a-5p */**, hsa-miR-23b-3p, hsa-miR-19a-3p */**, hsa-miR-21-5p */**, hsa-miR-20a-5p */**, hsa-miR-146a-5p, hsa-miR-27b-3p *, hsa-miR-4791, hsa-miR-31-3p */**, hsa-miR-33a-5p */**, hsa-miR-4284, hsa-miR-27a-3p *, hsa-miR-17-5p */**, hsa-let-7a-5p */**, hsa-miR-4321, hsa-miR-146b-5p **, hsa-miR-424-5p */**, hsa-miR-130a-3p, hsa-miR-30e-5p *, hsa-miR-30a-5p */**.	hsa-miRPlus-E1067 ebv-miR-BHRF1-2 hsa-miR-629 kshv-miR-K12-6-3p hsa-miRPlus-F1017.

MicroRNAs, the expression of which was impaired in lung cancer, are highlighted in bold. * MicroRNAs associated with the development of LUAD (lung adenocarcinoma). ** MicroRNAs associated with the development of LUSC (lung squamous cell carcinoma).

**Table 3:** The miRNAs that were significantly reduced after exposure to different concentrations of radon in cells.

Cellular Model of Irradiation	«2B-2.0 Gy (4)—20» [161]	«2B-2.0 Gy (4)—40» [161]	«Rn5-1 Cells (20,000 Bq/m3)» [160]	«Rn5-20 Cells (20,000 Bq/m3)» [160]
MicroRNAs whose profile has been altered by radioactive irradiation	hsa-miR-4730, hsa-miR-30c-2-3p */**, hsa-miR-6872-3p, hsa-miR-6132, hsa-miR-6798-3p, hsa-miR-634, hsa-miR-636 *.	hsa-miR-3907, hsa-miR-6732-3p, hsa-miR-4788.	hsa-let-7b-3p *, hsa-miR-625-3p */**, hsa-miR-877-5p */**, hsa-miR-4278, hsa-miR-3924, hsa-miR-3146, hsa-miR-4445-5p, hsa-miR-3660, hsa-miR-4516, hsa-miR-1249 **, hsa-miR-3191-5p, hsa-miR-4698, hsa-miR-193b-5p */**.	hsa-miR-4323, hsa-miR-4658, hsa-miR-1184, hsa-miR-4421, hsa-miR-144-5p */**, hsa-miR-32-3p **, hsa-miR-3685, hsa-miR-490-3p */**.

MicroRNAs, the expression of which was impaired in lung cancer, are highlighted in bold. * MicroRNAs associated with the development of LUAD (lung adenocarcinoma). ** MicroRNAs associated with the development of LUSC (lung squamous cell carcinoma).
